# The genetic and clinical characteristics and effects of Canakinumab on cryopyrin-associated periodic syndrome: a large pediatric cohort study from China

**DOI:** 10.3389/fimmu.2023.1267933

**Published:** 2023-09-21

**Authors:** Zhou Shu, Yue Zhang, Tongxin Han, Yan Li, Yurong Piao, Fei Sun, Jin Ma, Wenxiu Mo, Jiapeng Sun, Koon-Wing Chan, Wanling Yang, Yu-Lung Lau, Huawei Mao

**Affiliations:** ^1^ Department of Immunology, Ministry of Education, Key Laboratory of Major Diseases in Children, Beijing Children’s Hospital, National Center for Children’s Health, Capital Medical University, Beijing, China; ^2^ Department of Paediatrics and Adolescent Medicine, Li Ka Shing Faculty of Medicine, The University of Hong Kong, Hong Kong, Hong Kong SAR, China; ^3^ Beijing Key Laboratory for Genetics of Birth Defects, Beijing, China

**Keywords:** cryopyrin-associated periodic syndrome, genotype and phenotype correlation, novel mutation, Canakinumab, somatic mosaicism mutation

## Abstract

Cryopyrin-associated periodic syndrome (CAPS) comprises a group of disorders characterized by recurrent bouts of systemic inflammation related to overactivation of inflammasome. So far, neither large cases of the correlation between genotype and phenotype nor treatment strategies have been clearly stated in China. Here, we studied the clinical and genetic characteristics and their correlation from 30 CAPS patients in China. We identified the pathogenesis for novel mutations by activating *NLRP3* inflammasome for peripheral cells with ATP plus LPS, compared characteristics with other case series, and analyzed treatment outcomes of these patients. The patients harbored 19 substitutions in *NLRP3*, and 8 of them were novel mutations. Among these novel mutations, percentages of severe musculoskeletal, ophthalmologic, and neurological symptoms were higher compared with other case serials. The correlation of phenotypes and their variants seemed different in our cases, such as T350M, S333G/I/R, and F311V (somatic mosaicism). Ten patients received Canakinumab treatment, which proved effective at alleviating musculoskeletal, neurological, auditory, visual manifestations, fever, and rash for 10–20 months follow-up. Patients treated with prednisolone or prednisolone plus thalidomide or methotrexate, tocilizumab, TNF inhibiting agents, and sirolimus achieved only partial remission. Importantly, we firstly identified somatic mosaicism mutation of F311V, which was severe. Our study extended the spectrum of genotype and phenotype and characteristics of their correlations and provided detailed responses to different treatment strategies. These data provide guidance for future diagnosis and management for CAPS.

## 1 Introduction

Cryopyrin-associated periodic syndrome (CAPS) comprises a group of disorders with overlapping symptoms such as fever, urticarial rash, arthralgia, arthritis, and elevated acute-phase reactants (1–3). Previously, CAPS was classified into three subtypes according to distinct clinical characteristics. Familial cold autoinflammatory syndrome (FCAS, OMIM #120100) is the mildest form, presenting with paroxysmal fever, urticarial rash, and arthralgia, combined with conjunctivitis and headaches (4–8). Muckle–Wells syndrome (MWS, OMIM #191900) is the intermediate subtype, presenting with fever, urticarial rash, and systemic inflammation. Compared with FACS, arthralgia is often continuous in MWS, and arthritis is more common. In addition, patients can develop hearing loss, as well as renal failure due to amyloidosis, over time (4–6). The most severe subtype is neonatal-onset multisystem inflammatory disease (NOMID or CINCA, OMIM #607115). Apart from symptoms similar to FCAS and MWS, CINCA has special facial features such as frontal bossing, saddle-back nose, and a large cephalic perimeter. CINCA patients also develop central nervous system (CNS) disease, as well as early and progressive loss of hearing and vision due to inflammation. Approximately 50% of patients with CINCA, either treated or not, develop exophytic growth of the patella and epiphyses of the long bones (9). If CINCA is untreated during childhood, the mortality can be as high as 20% (9).

The clinical spectrum for CAPS is very broad and heterogeneous. Patients with mild and atypical symptoms were easy to misdiagnose. Because it has been recommended to make early diagnosis and provide proper treatment for CAPS, establishment of the characteristics associated with the clinical and genetic features of CAPS is of great importance. Until now, no large cases of the correlation between genotype and phenotype had been clearly stated in China, and little is known about the differences between Chinese pediatric/adult/Western patients. Thus, here we exhibited a cohort of 30 patients, summarized the clinical and genetic characteristics for Chinese patients, reviewed the literature, and made comparisons among them.

CAPS is caused by mutations in the *NLRP3* [nucleotide-binding oligomerization domain (NOD)-like receptor family, pyrin domain-containing 3] gene. CAPS is also known as cold-induced autoinflammatory syndrome 1 (CIAS1) (10). Gain-of-function mutations in *NLRP3* lead to a constitutive increase in secretion of interleukin-1 (IL-1) and IL-18, which is dependent on ASC and caspase 1 (11–13). Thus far, 262 mutations associated with CAPS have been documented in the INFEVER database (14). Previously published guidelines for genetic diagnosis of AID can guide physicians and geneticists (15). However, it is uncertain whether one mutation is strongly associated with specific clinical features. Likewise, little is known about its correlation with treatment responses.

Early diagnosis is crucial to initiate treatment before organ damage occurs. Kuemmerle-Deschner et al. proposed the following diagnostic criteria for CAPS in children and adults: elevated inflammatory markers (CRP and SAA) plus ≥2 of 6 typical CAPS signs or symptoms, which include (1) an urticaria-like rash, (2) cold/stress triggered episodes, (3) sensorineural hearing loss, (4) musculoskeletal symptoms (arthralgia/arthritis/myalgia), (5) chronic aseptic meningitis, and (6) skeletal abnormalities (epiphyseal overgrowth/frontal bossing) (16). Romano et al. reported EULAR/ACR points to be considered for diagnosis, management, and monitoring for CAPS (17). Regarding treatment, the European Medicines Agency (EMA) and Food and Drug Administration (FDA) allow three IL-1 blocking agents to be used for CAPS. Canakinumab was effective in two randomized controlled trials (RCTs) of patients with CAPS (18, 19). It was also effective in case series comprising all disease phenotypes and age categories (19–22). One case report showed that long-term usage of IL-1 blocking agents (starting at the age of 7 years, with the last follow-up visit at 20 years) helps to resolve femoral metaphyseal dysplasia (23). Although we conducted a review of the medical literature, we found little information about systemic detailed evaluation (skeletal, vision, hearing loss, and nervous system) in response to Canakinumab therapies and other treatment strategies for children in China.

Here, we summarized the genetic and clinical characteristics and the correlation of 30 CAPS patients in China. We identified novel mutations and compared the characteristics with pediatric, adult Chinese, and Western CAPS patients. We evaluated detailed responses of Canakinumab for 10 CAPS, and compared that with responses to other treatment strategies.

## 2 Materials and methods

### 2.1 Patients, clinical data, and literature review

Patients were either hospitalized or attending the outpatient department of Beijing Children’s Hospital, Department of Immunology. Patients were diagnosed, and monitoring strategies were set up, as described previously (5, 17, 24, 25). The patients were included in this study following diagnosis based on the criteria described above (16). Musculoskeletal manifestations were severe in cases of joint contractures, bone deformity, bone erosions, and osteolytic lesions and patellar overgrowth; without these, they were mild. Mild neurological involvement was defined by the presence of morning headache, papilledema, or aseptic meningitis, whereas severe neurological involvement was defined as the presence of seizures, hydrocephalus, or mental retardation. Mild ocular manifestations were defined by conjunctivitis or uveitis, whereas severe ocular involvement included optic nerve atrophy, cataract, glaucoma, and impaired vision (26). The diagnostic algorithm was shown in Ref (5). as follows: (1) suspected patients who presented with recurrent fever, headaches, musculoskeletal complaints, rash, especially increased irritability in children, and emotional lability in adults; (2) inflammatory makers including SAA and CRP examination; (3) differential diagnosis; and (4) genetic testing.

Clinical data and peripheral blood mononuclear cells (PBMCs) were collected. The study was performed in accordance with the Declaration of Helsinki and the ethics committee of Beijing Children’s Hospital (Beijing, China). All patients and/or guardians provided written informed consent to participate. Literature review used the same method reported (27), and Chinese reports were searched from January 2000 to May 2023.

### 2.2 Whole−exome sequencing, deep sequencing, and Sanger sequencing

Whole−exome sequencing (WES) was conducted on samples from patients with suspected CAPS. Genomic DNA was extracted from whole blood using the Axygen DNA Mini Kit (Axygen, China). A minimum of 3 μg of DNA was used for the indexed Illumina libraries (MyGenostics, China). Sanger sequencing was performed by MyGenostics to verify the mutation. Deep sequencing was also performed for patients with suspected CAPS, but for whom no *NLRP3* mutations were found by WES. HGMD and OMIM databases were searched for suspected variants. The pathogenicity of each variant was calculated according to ACMG criteria and the pathogenicity of the variants was speculated by Polyphen, SIFT, GERP++, and Mutation-Taster.

### 2.3 ELISA

For patients who carried novel suspected mutations, PBMCs were collected and frozen in liquid nitrogen as described above. PBMCs were stimulated for 4 h with lipopolysaccharide (LPS) (1 mg/mL; Sigma, USA) and adenine nucleoside triphosphate (ATP) (5 mM; Invivogen, USA). IL-1β and IL-18 levels in the cell supernatants were measured using ELISA kits according to the manufacturer’s instructions (BioLegend, USA; ExCell Biotech, China, respectively).

### 2.4 Flow cytometry analysis

T cells, B cells, and monocytes from P4 were sorted for deep sequencing further. PBMCs were stained with anti-CD3, anti-CD19, anti-CD14, and anti-CD16 antibodies (all from BioLegend, USA). Sorted cells were extracted using the Axygen DNA Mini Kit (Axygen, China). DNA sequencing was conducted as described above. PBMCs were stained with anti-CD3, anti-CD4, anti-CD8, anti-CD45 RA, anti-CD27, anti-CD19, anti-CD24, anti-IgD, anti-CD38, anti-CD16, anti-CD56, anti-β, and anti-γδ antibodies (all from BioLegend, USA).

### 2.5 Statistical analysis

Statistical analysis was conducted using GraphPad Prism software (version 8, GraphPad software). Data with a skewed distribution are expressed as the median and interquartile range (IQR, Q3–Q1). Cytokine data were assessed using an unpaired Student’s *t*-test. Fisher’s exact test was used for comparing frequencies of clinical manifestations by Statistical Product and Service Solutions (SPSS) 26 software; *p* < 0.05 indicates significance.

## 3 Results

### 3.1 Characteristics of patients

Thirty patients from 25 families were enrolled (12 with CINCA, 7 with MWS, and 11 with FCAS), namely, 18 male and 12 female patients (**Table 1**; **Figure S1**). The median age at onset was 0.25 years (IQR 0.82 years), and the median age at preliminary diagnosis was 3.25 years (IQR 6.5 years), with a median diagnostic delay of 2.34 years (IQR 5.3 years) (**Table 1**). P20–P22 and P23–P26 are from two families, respectively (**Table 1**; **Figure S1**), and other patients are from separate families.

**Table 1:** *NLRP3* gene mutations and general information.

Patients	Gender	Age at onset	Age at diagnosis	Nucleotide change	Amino acid	Exon	Subtypes	Status
P1	Male	1 day	1 Y	c.1336T > G	p. F446V	4	CINCA	Novel
P2	Female	2 days	4 M	c.1223T > C	p. M408T	4	CINCA	Ref. 2
P3	Male	3 days	6 M	c.1710G > T	p. K570N	4	CINCA	Novel
P4*	Male	2 days	4 Y 4 M	c.931T > G	p. F311V	4	CINCA	Novel
P5	Female	4 days	6 M	c.796C>T	p. L266F	4	CINCA	Ref. 7
P6	Male	2 days	8 M	c.1223T > C	p. M408T	4	CINCA	Ref. 2
P7	Male	2 days	1 Y	c.1703T>A	p. F568Y	4	CINCA	Novel
P8	Female	2 days	2 Y 1 M	c.796C>T	P. L266F	4	CINCA	Ref. 7
P9	Female	2 days	16 Y	c. 907G>T	p. D303N	4	CINCA	Ref. 26
P10	Male	2 days	5 Y	c. 998G>T	p.S333I	4	CINCA	Novel
P11	Male	2 days	38 Y	c.913G>A	p. D305N	4	CINCA	Ref. 41
P12	Male	2 days	1 M	c.1711G>C	p. G571R	4	CINCA	Ref. 7
P13	Male	8 M	3 Y 8 M	c.983G>A	p. G328E	4	MWS	Ref. 2
P14	Female	3 M	1 Y 4 M	−	−		MWS	−
P15	Male	3 Y	5 Y 8 M	−	−		MWS	−
P16	Female	2 Y	9 Y 4 M	c.430G > A	p. V144M	3	MWS	Novel
P17	Male	8 M	1 Y 4 M	c.1049C > T	p. T350M	4	MWS	Ref. 7
P18	Female	8M	11 Y	c.916G>A	p. E306K	4	MWS	Ref. 42
P19	Female	4 days	1 Y 4 M	c.1060G >A	p. A354T	4	MWS	Ref. 3
P20	Male	3 M	1 Y	c.1049C > T	p. T350M	4	FCAS	Ref. 7
P21	Female	6 M	35 Y	c.1049C > T	p. T350M	4	FCAS	Ref. 7
P22	Male	1 Y	61 Y	c.1049C >T	p. T350M	4	FCAS	Ref. 7
P23	Female	1 Y	2 Y 8 M	c.997A > G	p. S333G	4	FCAS	Novel
P24	Male	8 Y	26 Y	c.997A > G	p. S333G	4	FCAS	Novel
P25	Male	2 Y	20 Y	c.997A > G	p. S333G	4	FCAS	Novel
P26	Male	3 Y	58 Y	c.997A > G	p. S333G	4	FCAS	Novel
P27	Male	1 Y	3 Y 8 M	c.1049C>T	p. T350M	4	FCAS	Ref. 7
P28	Female	7 M	5 Y 7 M	c.784C > T	p.R262Ter	4	FCAS	Ref. 7
P29	Male	1 Y 4 M	2 Y 10 M	c.226G > A	p. A76T	2	FCAS	Novel
P30	Female	3 M	1 Y 8 M	c.1064T>C	p. L355P	4	FCAS	Ref. 7

Mutations are described with reference to GenBank sequences NM-004895. Mutations reported are labeled with a reference number. M: month; Y: year; (−) means gene negative after confirmation by whole exon sequencing. P4* indicates somatic mosaicism.

### 3.2 Characteristics of clinical phenotype

**Figure 1:**
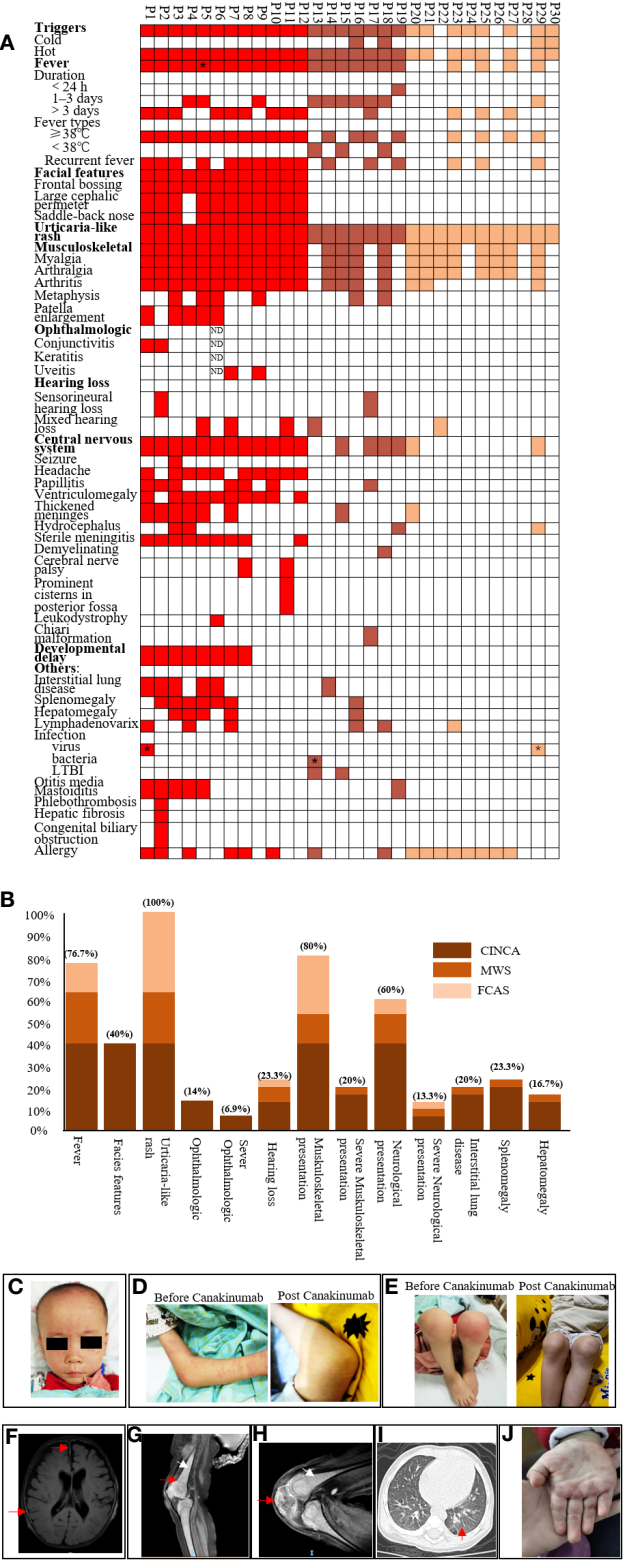
Phenotype summarization of cryopyrin-associated periodic syndrome patients. **(A)** shows phenotypes of each patient. Each column represents a patient, and each row represents a clinical manifestation. Red color indicates neonatal-onset multisystem inflammatory disease patients, brown color indicates Muckle–Wells syndrome patients, and yellow color indicates familial cold autoinflammatory syndrome patients. P5* indicates that P5 showed daily fever. P1* and P27* indicate that these patients had parainfluenza and influenza infection. P13* indicates that this patient had urinary tract infection with Gardnerella once; LTBI: latent tuberculosis infection.; **(B)** shows percentages of each clinical manifestation for three subtypes. Dark brown, light brown, and yellow indicate neonatal-onset multisystem inflammatory disease, Muckle–Wells syndrome, and familial cold autoinflammatory syndrome, respectively. **(C)** shows representative face features for neonatal-onset multisystem inflammatory disease (P1), frontal bossing, large cephalic perimeter, and saddle-back nose. **(D)** shows urticaria rash and no rash post-Canakinumab. **(E)** shows arthritis and patella enlargement, which seemed normal post-Canakinumab. **(F–H)** show features of MRI for CINCA patients; **(F)** shows thickened meninges (red arrow) and ventriculomegaly; **(G)** shows diffuse osteomyelitis (white arrow) and metaphysis overgrowth (red arrow); **(H)** shows patellar enlargement (red arrow); **(I)** shows interstitial lung disease for CAPS; and **(J)** shows other types of rash, like erythematous and edematous papules.

All patients exhibited systemic inflammation and an urticaria-like rash (**Figures 1A, B**). P14 also presented with erythema nodosum, which is rare (**Figure 1J**). In addition, 24 of 30 (76.7%) presented with fever, and 16 (53.3%) had recurrent fever. Among the 12 CINCA patients, 11 (91.7%) presented with fever, and 10 (83.3%) had recurrent fever. All 12 CINCA had special facial features such as frontal bossing, a large cephalic perimeter, and a saddle-back nose; the faces of other patients with CAPS appeared normal (**Figure 1C**). Among the 30 patients, 24 (80%) had musculoskeletal involvement, and 20% were severe. JADAS27 scores were 14.98 ± 9.6 before treatment (**Table S2**). Very specific features of CAPS, such as metaphysis overgrowth (**Figure 1G**), were observed mostly in patients with CINCA (4/12, 33.3%) and to a lesser extent in those with MWS (2/7, 28.6%). There were no such findings for FCAS. Five (16.7%) with CINCA showed patella enlargement (**Figures 1G, H**). Among 29 patients (1 patient refused to test), 4 (14%) had ocular disease, including conjunctivitis and uveitis; 2 had optic atrophy (P3 and P7). Of 30 patients, 7 (23.4%) had auditory disease: 2 had sensorineural hearing loss, 5 had mixed hearing loss (sensorineural hearing loss and obstructive hearing loss), and 7 had mild hearing loss (≥20 to ≤40 dB).

We found that 18 of 30 (60%) patients presented with neurological involvement and 13.3% were severe. P3, P4, P29, and P19 had hydrocephalus. P3 had seizures. Other symptoms included headache (33.3%), ventriculomegaly, thickened meninges, and sterile meningitis (30%) (**Figures 1A, B, F**). P6 had leukodystrophy. Two patients had cerebral nerve palsy (P7 and P11), which was rarely observed in CAPS. P11 experienced seven recurrent episodes of facioplegia; P11 also had prominent cisternae in the posterior fossa. P17 had a Chiari malformation (mild) that might not be associated with MWS.

Eight CINCA patients showed developmental delay. All presented with low body weight and shorter stature, but without intellectual disability. Six patients also had mild interstitial lung disease (P1, P2, P3, P5, P6, and P14) at preliminary diagnosis, which was a concomitant symptom with CAPS. Among the six patients, five patients were CINCA, and one patient was MWS. Some patients had allergic disease (food allergies or allergic rhinitis), noninfectious otitis media mastoiditis, splenomegaly, hepatomegaly, or lymphadenovarix caused by inflammation. P2 had a congenital biliary obstruction and showed mild hepatic fibrosis consistent with the pathology of liver biopsy taken in newborn. She also had phlebothrombosis of the right upper arm, possibly due to venipuncture. None had renal or gastrointestinal disease (**Figures 1A–I**).

### 3.3 Genetic features

**Figure 2:**
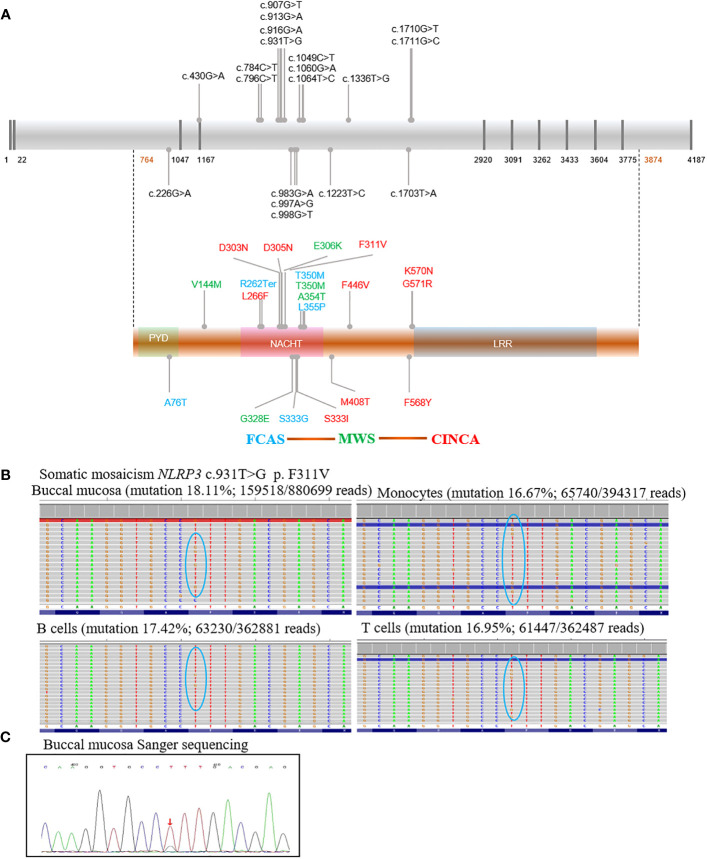
Schematic diagram of *NLRP3* mutations in our cohort and somatic mosaicism of P4. All potential mutations associated with cryopyrin-associated periodic syndrome were shown in the schematic diagram. The presence of multiple mutations coding the same amino acid suggested mutational hotspots. There was fairly consistent genotype–phenotype correlation indicated by colors: Familial cold autoinflammatory syndrome (blue), Muckle–Wells syndrome (green), Neonatal onset multisystemic inflammatory disease (red) **(A)**. Mutation was described with reference to GenBank sequences NM-004895 and NP_004886. **(B)** shows mutation rates for P4, in whom somatic mosaicism was identified by high depth sequencing. **(C)** shows verification for P4 by Sanger sequencing; familial cold autoinflammatory syndrome (FCAS), Muckle–Wells syndrome (MWS), Neonatal onset multisystemic inflammatory disease (NOMID, CINCA), nucleotide oligomerization domain (NOD), and Leucine rich repeat (LRR). ↓ T>G mutation.

**Figure 3:**
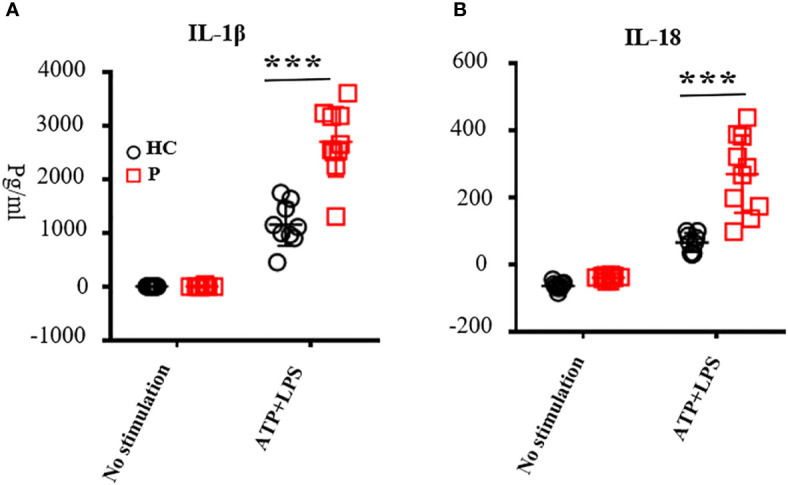
The levels of IL-1β **(A)** and IL-18 **(B)** in the supernatant secreted by peripheral blood mononuclear cells (PBMCs). PBMCs were stimulated with adenine nucleoside triphosphate and lipopolysaccharide. Supernatant was collected to test the levels of IL-1β and IL-18 by ELISA according to the manufacturer’s guidelines; nine healthy controls were tested. Patients included P1, P3, P4, P7, P13, P16, P19, P22, P25, and P28; the number of patients was 10 in total. ATP, adenine nucleoside triphosphate; LPS, lipopolysaccharide; PBMCs, peripheral blood mononuclear cells; ***, <0.001. HC, healthy control; P, patient.

As shown in **Table 1**; **Figure 2A**, patients carried 19 substitutions, and 8 of them were newly discovered (F446V, F311V, S333I, S333G, V144M, A76T, K570N, and F568Y). Most of them were located in exon 4, in the NATCH domain (**Figure 2A**). The ACMG criteria, Polyphen, SIFT, GERP++, and Mutation-Taster for novel mutations are shown in **Table S1**. Functional verification experiments were done for novel mutations except for P10 (S333I), because we did not receive a sample from P10 (**Figures 3A, B**). Both Polyphen and SIFT showed damaging properties, Mutation-Taster showed disease-causing properties, and GERP++ was conserved for S333I substitution (**Table 1**; **Table S1**). Combined with the clinical feature shown in **Figure 1A**, we thought P10 carried mutation causing CINCA. All patients with novel mutations had CAPS manifestation and were clearly differentiated from other diseases (**Figure 1A**). Two patients in this study underwent WES and were *NLRP3* gene negative. They were diagnosed because they presented with CAPS symptoms and were excluded from other diseases. We thus suspected that these patients may have a somatic mosaicism; however, the parents refused deep sequencing for further investigation (**Table 1**).

### 3.4 Somatic mosaicism identification and genotype–phenotype correlation analysis

Mutations and related subtypes of CAPS are shown in **Figure 2A**. P4, with a presentation of typical CINCA, harbored no mutations while WES was performed twice at different hospitals. Deep sequencing identified a somatic mosaicism (*NLRP3*: c.931T>G, p. F311V), with mutation rates of 18.11%, 16.67%, 17.42%, and 16.95% in the buccal mucosa, monocyte, B cells, and T cells, respectively (**Figure 2B**). Sanger sequencing was performed for verification (**Figure 2C**). Five patients from two families carried the T350M mutation, while only P17 and P22 had hearing loss. Four CAPS patients from a family carried the S333G mutation, presented with the milder MWS or FCAS phenotypes. P23 and P25 had recurrent fever, and P24, P25, and P26 had myalgia and arthralgia. P5 and P8 from two families carried the L266F mutation and presented with CINCA. Two CINCA patients carried the M408T mutation.

### 3.5 Laboratory data and immunological characteristics

For all patients carrying novel mutations and some other patients reported previously, we performed functional studies. Levels of IL-1β and IL-18 secreted by PBMCs upon LPS and ATP stimulation from patients were increased compared with healthy controls (*n* = 10; P1, P3, P4, P7, P13, P16, P20, P23, P26, and P29; **Figures 3A, B**). Most patients showed increased serum IL-6 levels. Only some of the patients exhibited increased IL-1β in the serum, probably because we did not test these during flares (**Table S2**). All patients showed high levels of inflammatory markers at the time of diagnosis. The absolute number of white blood cells, neutrophil, CRP, ESR, and SAA increased above normal levels (**Table S2**). When comparing CINCA with other types of CAPS, we found no significant difference in WBC, CRP, and ESR (*n* = 25, **Table S3**). To investigate whether *NLRP3* mutations affect lymphocyte subsets, we analyzed the absolute number of lymphocytes (*n* = 10; CD3+T cells, CD4+T cells, CD4/CD8 naïve, CD4/CD8 CM, CD4/CD8 EM, CD4/CD8 TEMRA, CD19+B, Naïve, PBC, MBC, NK, DNT, and γδT cells), as described previously (28). The results were almost normal (**Table S4**). JADAS27 scores were recorded at every follow-up visit. At the time of diagnosis, the JADAS27 scores were 18.6 ± 8.4 for CINCA (*n* = 12) and 13.4 ± 6.7 for other CAPS (*n* = 8) (**Table S3**).

### 3.6 Characteristics for novel mutations and comparison with previous reports

The reported CAPS were less than 100 until now in China (26, 27, 29–35). The number of reported cases is very limited compared with the large number of potential CAPS patients in China. As shown in **Table 2**, we compared the characteristics of our novel mutations (*n* = 11) with Chinese pediatric (*n* = 72, including pediatric patients in our study), adult (*n* = 25), and Western patients (*n* = 136). Eleven patients with novel mutations showed recurrent fever, urticarial rash, and musculoskeletal involvement. Among novel mutations, K570N, F311V, and A76T showed severe neurological involvement, as they had hydrocephalus tested by MRI. F446V, K570N, and F311V showed patella enlargement, which indicated severe symptoms for bone. K570N and F568Y had optic atrophy, which also indicated severe symptoms in eyes. Among the patients with novel mutations, severe musculoskeletal, ophthalmologic, and neurological symptoms were 36.4%, 18.2%, and 27.3%, respectively, which were high. Combining these data together, Chinese pediatric patients showed higher percentage of fever, rash, and neurological involvement compared with Chinese adult patients. Chinese pediatric patients also exhibited higher percentage of fever, neurological and severe musculoskeletal involvement, and lower total musculoskeletal or ophthalmologic involvement compared with Western patients (**Table 2**).

**Table 2:** *NLRP3* novel mutations and comparison with pediatric CAPS, pediatric compared with adult Chinese, and Western CAPS.

Clinical feature		Novel mutations							A	B	C	D	P1 (A vs. B)	P2 (B vs. C)	P3 (B vs. D)	P4 (A vs. D)
	F446V	F311V	S333I	S333G	V144M	A76T	K570N	F568Y	Summary (*n* = 11)	Pediatric Chinese (*n* = 72)	Adult Chinese (*n* = 25)	Western (*n* = 136)				
Fever	Yes	Yes	Yes	Yes	Yes	Yes	Yes	Yes	9/11 (81.8%)	66/72 (91.7%)	20/24 (83.3%)	95/136 (70%)	0.286	0.0144	0.0001	0.51
Rash	Yes	Yes	Yes	Yes	Yes	Yes	Yes	Yes	11/11 (100%)	69/72 (95.8%)	20/24 (83.3%)	132/136 (97%)	1	0.025	0.695	1
Musculoskeletal	Yes	Yes	Yes	Yes	Yes	Yes	Yes	Yes	10/11 (90.9%)	48/72 (66.7%)	16/24 (66.7%)	117/136 (86%)	0.16	0.811	0.002	1
Severe musculoskeletal	Yes	Yes	No	No	Yes	No	Yes	No	4/11 (36.4%)	(10/72, 13.9%)	not sure	6/136 (4%)	0.084		0.026	0.003
Ophthalmologic	Yes	No	No	No	No	No	Yes	Yes	2/11 (18.2%)	24/71 (33.8%)	4/24 (16.7%)	97/136 (71%)	0.489	0.127	0.0001	0.001
Severe ophthalmologic	No	No	No	No	No	No	Yes	Yes	2/11 (18.2%)	Not sure	2/24 (8.3%)	16/136 (12%)				0.626
Hearing loss	No	No	No	No	No	No	No	Yes	1/11 (9.1%)	24/72 (33.3%)	7/24 (29.2%)	56/136 (42%)	0.16	0.804	0.297	0.051
Neurological	Yes	Yes	Yes	No	No	Yes	Yes	Yes	6/11 (54.5%)	40/72 (55.6%)	7/20 (35%)	55/136 (40%)	1	0.021	0.041	0.526
Severe neurological	No	Yes	No	No	No	Yes	Yes	No	3/11 (27.3%)	10/72 (13.9%)	2/20 (10%)	16/136 (12%)	0.366	0.725	0.664	0.154

Q705K did not describe other symptoms except for neurological involvement (29). K129R, Q703K, V70M, P38S, and D29V did not describe neurological involvement, so they were excluded from the denominator (30). Fisher exact test was used for comparing frequencies of clinical manifestations by Statistical Product and Service Solutions (SPSS) 26 software; p value < 0.05 means significant. P1: novel mutations vs. pediatric Chinese patients; P2: pediatric Chinese patients vs. adult Chinese patients; P3: pediatric Chinese patients vs. Western patients; P4: novel mutations vs. Western patients.

### 3.7 Treatment and outcome

**Figure 4:**
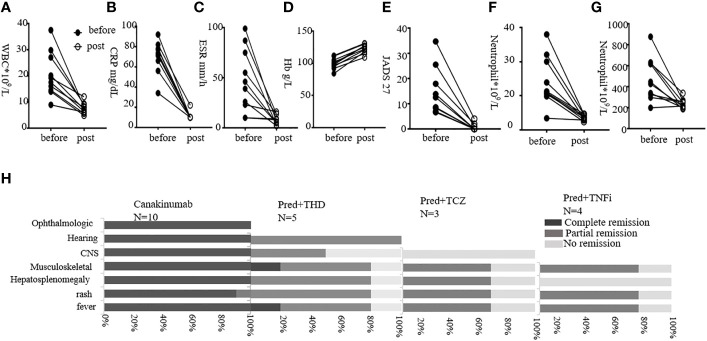
Treatment outcomes following different strategies. **(A–G)** show levels of WBC, CRP, ESR, JADAS 27 score, neutrophil, and platelet before and post-Canakinumab treatment, respectively. **(H)** shows responses post-Canakinumab for each manifestation. Percentages of remission are shown. Pred, prednisone; TCZ, tocilizumab; THD, thalidomide; TNFi, tumor necrosis factor inhibiting agents.

In this study, four patients received prednisolone with adalimumab (Ada) (one changed to Infliximab 3 months later), and three patients received prednisolone with tocilizumab (TCZ). Four patients received prednisolone with sirolimus. Ten patients received prednisolone with thalidomide (THD) (five lost to follow-up, so five were summarized) (**Figure 4H**; **Tables S5**, **S6**). Among four patients with adalimumab treatment, three (75%) experienced partial remission of fever, acute reactants, flares, rash, and musculoskeletal disease. These patients had no vision and hearing loss, or CNS involvement, so we lacked data for responses to these symptoms. Among three patients with tocilizumab treatment, two patients (66.7%) experienced partial remission of fever, rash, musculoskeletal manifestation, and hepatosplenomegaly. P3 with TCZ had no remission of CNS symptoms. Among five patients with THD, partial plus methotrexate together, three (60%) showed partial febrile improvement and relief of joint symptoms. Four of the five (80%) experienced remission of the rash and hepatosplenomegaly. Of these, two had CNS disease, and one got partial remission. Among THD treatment CAPS, only P17 had hearing loss. He recovered from hearing loss, rash, fever, and neurological manifestation after 1.5 years of thalidomide treatment. However, one of the five (P5, 20%) got worse and presented with daily fever. Also, the JADAS scores increased from 34 to 42.

Here, we gave Canakinumab to 11 patients and followed up 10 of them. The dosage was 4 mg/kg, given at 8-week intervals. An exception was P21 (the mother of P20), who received a single dose of 150 mg. The youngest patient to receive Canakinumab was 1 year old. The average follow-up was 12.9 months (range, 8–20 months). Patients were followed up every 2 months in the outpatient department, and laboratory examinations were performed (**Figures 4A–H**).

For 10 patients, the urticaria rash disappeared quickly after Canakinumab treatment, with the fastest time being 3 h post-injection. P14 still showed erythematous and edematous papules after Canakinumab treatment, and those symptoms disappeared after THD was added for 4 months. The WBC, CRP, and ESR returned to normal, as did anemia, very soon post-treatment. An exception was P2, who had typical CINCA symptoms. Therefore, we increased the dosage to 6 mg/kg given at 8-week intervals. However, P2 still had fluctuating inflammatory marker levels. After her Canakinumab treatment was shortened to 4-week intervals, P2 entered remission. Among the 10 patients, musculoskeletal disease was alleviated post-treatment. The JADAS27 scores fell to normal in all patients except P1 and P16, whose scores still fell markedly but remained a little above normal. Overall, Canakinumab was very effective in improving patella enlargement, hearing, vision, and CNS disease. We found that hearing loss returned to normal in P13 and P2 after 6–12 months of Canakinumab treatment. In addition, vision improved in P1, P2, and P3 post-treatment. P1–P4 had no headache, and CSF analysis was normal. Brain MRI should be repeated to evaluate iconography variations in the future. Moreover, four patients with interstitial lung disease recovered after 3–6 months of Canakinumab treatment.

## 4 Discussion

Here, we describe the clinical phenotypes, genetic features, immunological characteristics, and treatment of 30 pediatric patients with CAPS in China. Consistent with previous studies (36), we found that almost all CINCA patients showed disease onset immediately after birth, and most (25 of 30) experienced onset at younger than 1 year of age, suggesting that early onset is a strong characteristic of CAPS. In our study, the median age at diagnosis was 1.6 years, which is one of the possible reasons that we noted organ damage. The latest onset age in our cohort was 8 years, and the oldest diagnostic age was 58 years; the oldest patient had mild symptoms. This patient was identified because the youngest granddaughter experienced onset at 1 year. Thus, we should not exclude elder adults, especially in milder cases. We should also think thoroughly about untypical cases.

In addition, the clinical features were summarized. The common symptoms that occurred for Chinese CAPS were consistent with adult and Western CPAS. However, specific symptom prevalence of our cases differed from reported, and we had rare symptoms such as recurrent episodes of facioplegia and erythema nodosum. The patients with CINCA had more severe fever than those with other forms of CAPS; fever was of longer duration and had a higher peak temperature. Consistently, CINCA was more severe as reported (37–39). Similarly, the incidence of musculoskeletal involvement was as high as 80%, and myalgia and arthralgia were seen in all subtypes. Arthritis was seen in those with MWS and CINCA but not in those with FCAS. Very specific features of CAPS, metaphysis overgrowth and patella enlargement, were common in those with CINCA. These were distinguished from arthritis (not single gene disease). Studies report corneal involvement in 40% of CAPS cases. Uveitis can affect 50% of CINCA patients, and more than 80% have an affected optic nerve head (37–39). Here, we found that 27.6% of patients had ocular disease, and 58.3% of CINCA patients had papillitis; these percentages are lower than reported in other studies, possibly because we included only 12 CINCA patients in our cohort. Patients with MWS and CINCA show progressive sensorineural hearing loss caused by degeneration of sensoric structures in the organ of Corti (40). In our study, some patients presented with sensorineural and obstructive mixture hearing loss, probably due to recurrent otitis media mastoiditis. Another devastating and specific feature for CINCA is CNS impairment. Aseptic meningitis, brain atrophy, and enlarged ventricles are common (10). Consistent with this, our CINCA patients showed a high incidence of aseptic meningitis (75%), ventriculomegaly (83.3%), and thickened meninges (50%). They also showed rare features such as cerebral nerve palsy, Chiari malformation, leukodystrophy, and prominent cisternae in the posterior fossa. By combining cases together, we found that pediatric Chinese cases seemed more severe than those of Western patients. Chinese pediatric patients exhibited higher percentage of fever, neurological and severe musculoskeletal involvement, and lower total musculoskeletal or ophthalmologic involvement compared with Western patients. Furthermore, we found that the specific symptom prevalence of Chinese pediatric patients was similar except for higher percentage of fever, rash, and neurological involvement. These indicate that ethnic population was probably one of the factors affecting CAPS pathogenesis. In the future, we need to collect more cases to compare the difference between pediatric and adult CAPS, to provide support for treatment and long-term follow-up emphasis.

Furthermore, we identified eight novel mutations and extended the recognition of genotype with phenotype correlation, which had some differences in our case compared with reported cases. To date, approximately 262 different sequence variants of the *NLRP3* gene associated with a CAPS phenotype have been identified (14, 41, 42). Here, we found 19 substitutions, and the substitutions were mainly located in exon 4, which were very similar as reported. Among eight novel mutations, K570N, F311V, and A76T showed hydrocephalus (severe neurological involvement). F446V, K570N, and F311V exhibited patella enlargement (severe in bone). K570N and F568Y exhibited optic atrophy (severe in eyes). All these were consistent with reporting that rare variants presented with a more severe phenotype (26).

F311V was somatic mosaicism in our case, which was different from somatic mosaicism mutation features reported previously. Genotype-matched comparison confirmed that patients with somatic mosaicism presented with milder neurologic symptoms and lower incidence of intellectual disability (43). However, F311V exhibited hydrocephalus and patella enlargement, which was more severe. In addition, the reported late-onset somatic *NLRP3* mosaicism was consistent with mild spectrum of CAPS presentations (44). All these suggested that somatic *NLRP3* mosaicism could be mild or severe. Doctors should suspect CAPS whether patients were mild or severe, early onset or late onset.

In our case, four patients carried S333G and one patient carried S333I, both of which were novel. S333G patients presented with FCAS phenotype, but S333I exhibited CINCA, and S333R showed CINCA (45). T350M was highly associated with hearing loss (26). There were five patients carrying the T350M mutation in our case, of whom only two had mild hearing loss, and one of the two developed hearing loss in his fifties. D305N was highly associated with severe phenotype (26). The patient who had the same mutation in our case was consistent. Two patients had L266F in our case, and they exhibited CINCA as reported (9). Combined together, depending on the analysis variants with phenotype, it seemed that factors other than genetics, such as ethnic population, could affect pathogenesis of CAPS. The picture is more complicated than we first thought, and more investigations are needed.

CAPS is a very heterogeneous disease, meaning that stratified individual treatment approaches are needed urgently to prevent organ damage. There are consensus or recommendations for CAPS diagnosis, management, and monitoring (5, 17, 24, 25). Although Canakinumab was effective, China lacks data regarding responses to Canakinumab in children. In addition, reports about patients who cannot afford Canakinumab treatment are limited. By analyzing the responses to prednisolone or prednisolone plus thalidomide, tocilizumab, TNF inhibiting agents, and sirolimus, respectively, it seemed that sirolimus was of no benefit for CAPS. Prednisolone add-on tocilizumab, tumor necrosis factor inhibiting agents, and thalidomide resulted in partial remission, mainly for acute reactants phase, rash, and musculoskeletal manifestations. However, partial patients had no responses. Surprisingly, P17 recovered hearing after 1.5 years of treatment with prednisolone plus thalidomide. Overall, Canakinumab was effective for our 10 CAPS patients followed up for 10–20 months. It appeared to improve inflammation, rash, musculoskeletal disease (including metaphysis overgrowth and patella enlargement), hearing, vision, and CNS disease. Because our patients had specific characteristics, we will investigate whether Canakinumab improves cranial nerve palsy by long-term follow-up. The main study limitation was the small cohort; we need to recruit more patients to validate these findings. Also, the follow-up was not long term, making it difficult to analyze changes in iconography; therefore, the follow-up period should be extended.

We reported two patients, presenting with typical CAPS clinical features, who were *NLRP3* gene negative. They fulfilled the diagnostic criteria described above. We suspected *NLRP3* somatic mosaicism; however, the patients refused to undergo deep sequencing to verify. This was one limitation in our study. Another limitation was that we lacked long-term Canakinumab treatment response data, as the longest follow-up period was 20 months. Moreover, this was a single-center study, and most patients were pediatric patients, who lacked adult CAPS characteristics.

## 5 Conclusion

This study summarized the clinical and genetic characteristics of 30 CAPS patients in China, and extended partial correlation of the phenotype with variants. We enriched the pathogenesis of novel mutations and improved partial recognition for CAPS. We identified patients with somatic mosaicism who presented with severe phenotype; thus, we recommended the somatic mosaicism test for *NLRP3* gene-negative patients whether their clinical phenotypes were severe or mild. Importantly, our study will potentially help doctors to achieve better diagnosis and management on CAPS.

## Data availability statement

The data presented in the study are deposited in the China National GeneBank Databases with the accession number of CNP0004750.

## Ethics statement

The studies involving humans were approved by Ethics committee of Beijing Children’s Hospital. The studies were conducted in accordance with the local legislation and institutional requirements. Written informed consent for participation in this study was provided by the participants’ legal guardians/next of kin. Written informed consent was obtained from the individual(s), and minor(s)’ legal guardian/next of kin, for the publication of any potentially identifiable images or data included in this article.

## Author contributions

ZS: Conceptualization, Data curation, Formal Analysis, Writing – original draft. YZ: Writing – review & editing. TH: Writing – review & editing. YL: Writing – review & editing. YP: Writing – review & editing. FS: Writing – review & editing. JM: Writing – review & editing. WM: Writing – review & editing. JS: Writing – review & editing. K-WC: Writing – review & editing. WY: Writing – review & editing. YLL: Writing – review & editing. HM: Writing – review & editing, Funding acquisition.
