# Supplementary material for: The genetic and clinical characteristics and effects of Canakinumab on cryopyrin-associated periodic syndrome: a large pediatric cohort study from China
Source: Front Immunol. 2023 Sep 21;14:1267933. doi: 10.3389/fimmu.2023.1267933 (PMC10551459; doi:10.3389/fimmu.2023.1267933)
Supplement: Supplementary file 1 [file DataSheet_1.docx]

Table S1 The ACMG criteria, Polyphen, SIFT, GERP++ and Mutation-Taster for novel mutations.

| Novel Mutations | ACMG | Polyphen | SIFT | GERP++ | Mutation-Taster | Frequency  In database |
| --- | --- | --- | --- | --- | --- | --- |
| F446V | Uncertain | Possibly damaging | Damaging | Conserved | Disease causing | - |
| F311V | Uncertain | Benign | Damaging | Conserved | Disease causing | - |
| S333I | Uncertain | Possibly damaging | Damaging | Conserved | Disease causing | - |
| V144M | Uncertain | Possibly damaging | Damaging | Non-conserved | Polymorphism | 0.0000039 |
| A76T | Uncertain | Tolerated | Benign | Non-conserved | Polymorphism | 0.00039 |
| K570N | Likely pathogenic | Benign | Damaging | Benign | Disease causing | - |
| F568Y | Uncertain | Possibly damaging | Tolerated | Conserved | Polymorphism | - |
| S333G | Uncertain | Benign | Benign | Conserved | Polymorphism | - |

Frequency database: 1000g2015aug_al;

Table S2. Laboratory examination of cryopyrin-associated periodic syndrome (before treatment)

|  | Regular blood test | | | | | | | Serum cytokines | | | | | | |  | |
| --- | --- | --- | --- | --- | --- | --- | --- | --- | --- | --- | --- | --- | --- | --- | --- | --- |
| Patients | WBC  *10^9^/L | Platelets  *10^9^/L | Hb  g/L | CRP  mg/L | ESR  mm/h | SAA  mg/L | IL-1β  pg/ml | | IL-1RA  pg/ml | IL-18  pg/ml | IL-6  pg/ml | TNF-α  pg/ml | IFN-γ  pg/ml | JADAS27 | |  |
| P1 | 13.97 | 624 | 92 | 71 | 87 | 197 | N | | 6195 | N | 36.91 | N | 92.6 | 34.7 | |  |
| P2 | 18.39 | 426 | 97 | 82 | 23 | 89.2 | N | | N | N | 8.89 | N | 23.42 | 8.3 | |  |
| P3 | 30.41 | 616 | 84 | 141 | 38 | 76.8 | 26.3 | | N | N | 2466 | N | N | 13.8 | |  |
| P4 | 27.08 | 876 | 95 | 73.6 | 55 | ND | N | | N | N | 143.9 | N | N | 12.5 | |  |
| P5 | 21.98 | 152 | 70 | 73.3 | 126 | 168 | 33.4 | | 5233 | 1355 | 55.6 | N | N | 34 | |  |
| P6 | 11.7 | 431 | 78 | 73 | 40 | 244 | 14.44 | | ND | N | 7.68 | N | N | 19.3 | |  |
| P7 | 30.5 | 898 | 82 | 199 | 156 | 335 | N | | N | N | 141.3 | N | N | 22 | |  |
| P8 | 22.3 | 464 | 99 | 56 | 22 | ND | ND | | ND | ND | ND | ND | ND | 12.2 | |  |
| P9 | 18.9 | 532 | 104 | 109 | 96 | ND | ND | | ND | ND | ND | ND | ND | 18.6 | |  |
| P10 | 21.11 | 648 | 91 | 112 | 45 | ND | ND | | ND | ND | ND | ND | ND | 12.5 | |  |
| P11 | 28.89 | 234 | 84 | 156 | 103 | ND | ND | | ND | ND | ND | ND | ND | 21.3 | |  |
| P12 | 16.55 | 921 | 75 | 100 | 56 | ND | ND | | ND | ND | ND | ND | ND | 13.6 | |  |
| P13 | 26.6 | 446 | 114 | 36 | 25 | 91.6 | N | | 5449 | N | N | N | N | ND | |  |
| P14 | 14.51 | 325 | 105 | 66 | 75 | 79.8 | N | | N | N | 89.3 | N | N | 25.5 | |  |
| P15 | 8.9 | 199 | 103 | 34 | 39 | 68.9 | N | | N | N | 29.7 | N | N | 6.9 | |  |
| P16 | 17.48 | 631 | 95 | 92 | 99 | 189 | 13.7 | | 2557 | N | 97.4 | 24.1 | N | 17.9 | |  |
| P17 | 15 | 379 | 124 | 33.5 | 22 | 28.3 | 14.1 | | N | N | 7.03 | N | N | ND | |  |
| P18 | 22.35 | 380 | 104 | 70 | 34 | 197 | N | | N | N | 19.4 | N | N | 16.4 | |  |
| P20 | 16.04 | 297 | 100 | 77 | 26 | 178 | N | | 3562 | 1988 | 98.7 | N | N | 6.6 | |  |
| P23 | 28.22 | 434 | 109 | 216 | 156 | 296 | 14.9 | | ND | N | 57.6 | N | N | 6.6 | |  |
| P27 | 15.9 | 432 | 105 | 89 | 56 | 143.6 | 15.9 | | N | N | 119 | 9.77 | N | 11.6 | |  |
| P28 | 17.8 | 562 | 99 | 167 | 89 | 156 | ND | | ND | ND | ND | ND | ND | ND | |  |
| P29 | 20.63 | 322 | 109 | 20 | 34 | 76.9 | N | | N | N | 87.4 | N | 23.42 | 15.4 | |  |
| P19 | 35 | 800 | 91 | 33 | 22 | 947 | N | | N | 4566 | N | N | N | ND | |  |
| P30 | 21.8 | 677 | 105 | 56 | 34 | 68.9 | N | | N | N | 46.9 | N | N | ND | |  |

Blood was collected in the hospital. Serum cytokines were analyzed using Elisa Kits. Some patients were not scored with JADAS27 because they didn’t have arthritis. And we labeled with “not done, ND”. Normal ranges: IL-1β ≤ 12.4 pg/ml; IL-1RA ≤ 2171 pg/ml; IL-18 ≤ 957 pg/ml; IL-6 ≤ 5.4 pg/ml; TNF-α ≤ 3.1 pg/ml; IFN-γ ≤ 4.5 pg/ml.

WBC: white blood cells; Hb: hemoglobin; CRP: C-reactive protein; ESR, erythrocyte sedimentation rate; SAA: serum amyloid A; IL-1β: interleukin-1β; IL-1RA: interleukin-1 receptor antagonist; IL-18: interleukin-18; IL-6: interleukin-6; TNFα: tumor necrosis factor α; IFN-γ: interferon-γ; N: normal; ND: not done; NA: not available.

Table S3. Comparison of inflammatory markers and JADAS 27 scores between CINCA and other types of CAPS before treatment

|  | CINCA（X ± SD） | Other （X ± SD） | P-value |
| --- | --- | --- | --- |
| WBC (×10^9^/L) | 21.8 ± 6.3 | 20.0 ± 7.2 | P = 0.51 |
| CRP mg/L | 103.8 ± 42.5 | 76.1 ± 57.0 | P = 0.18 |
| ESR mm/h | 70.6 ± 42.7 | 54.7 ± 40.0 | P = 0.35 |
| JADAS 27 score | 18.6 ± 8.4 | 13.4 ± 6.7 | P = 0.14 |

CINCA: neonatal-onset multisystem inflammatory disease; WBC: white blood cells; CRP: C-reactive protein; ESR, erythrocyte sedimentation rate; X ± SD: Average± standard deviation; P≤0.05 means significant;

Table S4. Immunological tests for CAPS

|  |  |  | CD4 subsets numbers | | | | | CD8 subsets numbers | | | | | CD19 subsets numbers | | | | | NK | | γδT | | DNT | |
| --- | --- | --- | --- | --- | --- | --- | --- | --- | --- | --- | --- | --- | --- | --- | --- | --- | --- | --- | --- | --- | --- | --- | --- |
| Patients |  | CD3 /µL | CD4 /µL | Naïve /µL | CM /µL | EM /µL | TEMRA /µL | CD8 /µL | Naïve /µL | CM /µL | EM /µL | TEMRA /µl | CD19 /µl | Naïve /µL | MBC /µL | PBC /µL | /µL | | /µL | | /µL | |  |
| P1 |  | 2242 | 1136 | 729 | 382 | 25 | 0 | 858 | 668 | 58 | 23 | 9 | 707 | 595 | 48 | 12 | 167 | | 279 | | 83 | |  |
| P3 |  | 5640 | 2703 | 1881 | 584 | 168 | 3 | 2743 | 1495 | 321 | 573 | 354 | 1572 | 1335 | 97 | 6 | ND | | 171 | | 63 | |  |
| P4 |  | 3470 | 1745 | 1084 | 511 | 147 | 3 | 1544 | 928 | 119 | 151 | 346 | 685 | 440 | 124 | 11 | 647 | | 121 | | 50 | |  |
| P13 |  | 3270 | 1670 | 902 | 698 | 68 | 2 | 1244 | 597 | 372 | 174 | 100 | 1261 | 608 | 192 | 62 | 167 | | 279 | | 83 | |  |
| P14 |  | 2282 | 1428 | 847 | 560 | 19 | 3 | 712 | 622 | 79 | 8 | 4 | 1514 | 385 | 103 | 6 | 89 | | 62 | | 41 | |  |
| P15 |  | 2353 | 1158 | 823 | 248 | 79 | 8 | 815 | 595 | 148 | 37 | 34 | 582 | 314 | 161 | 0 | 226 | | 325 | | 21 | |  |
| P16 |  | 3012 | 1565 | 1158 | 351 | 41 | 17 | 1288 | 845 | 164 | 146 | 134 | 672 | 577 | 60 | 5 | 124 | | 133 | | 32 | |  |
| P17 |  | 2459 | 1290 | 951 | 315 | 23 | 1 | 941 | 784 | 129 | 8 | 20 | 1028 | 6 | 86 | 3 | 413 | | 172 | | 27 | |  |
| P18 |  |  | 696 | 267 | 337 | 84 | 8 | 459 | 228 | 151 | 44 | 37 | 333 | 8 | 67 | 6 | 108 | | 86 | | 27 | |  |
| P29 |  | 3216 | 1857 | 1361 | 468 | 22 | 4 | 1110 | 969 | 99 | 17 | 26 | 852 | 559 | 112 | 2 | ND | | 262 | | 34 | |  |

The absolute numbers of different subsets were analyzed by flow cytometry. CM: central memory; EM: effector memory; DNT: αβ+ double negative T cells; TEMRA: End-differentiated effector memory cells.

Red denotes increased numbers. Green denotes reduced numbers. We used the same methods in all cases, and assessment of absolute numbers was made according to reference values for lymphocyte numbers among different age groups in China (27).

Table S5. Treatments and outcomes of patients before and after Canakinumab

| Patient | Treatment | Treatment |  | Follow-up | | Outcome (last visit; 4mg/kg, 8 week intervals) | | | | | |
| --- | --- | --- | --- | --- | --- | --- | --- | --- | --- | --- | --- |
|  | Strategy | Course |  | Time | Laboratory data | | Organ specific | Hearing | Vision | CNS | Musculoskeletal |
| **P1**  **Before** | Pred+THD+MTX | 1 M |  | 1 M | WBC (13.97)  CRP (71)  ESR (87)  Hb (92) | | Fever;  Rash;  Mastoiditis | Normal  (-10–20 dB) | Conjunctivitis;  Papillitis (mild) | Headache;  Thickened meninges;  Ventriculomegaly | Myalgia;  Arthralgia;  Arthritis (JADAS27: 34.7);  Joint contraction (knees);  Patella enlargement; |
| **P1**  **Post** | Pred+MTX  +Cana | 10 M |  | 10 M | WBC (4.84)  CRP (＜10)  ESR (14)  Hb (109) | | Normal | Normal  (-10–20 dB) | Normal | No headache;  Thickened meninges alleviated;  Ventriculomegaly alleviated; | (JADAS27: 3);  Joint contraction alleviated (knees) |
| **P2* Before** | Pred+ sirolimus | 8 M |  | 8 M | WBC (19.25)  CRP (82)  ESR (23)  Hb (97) | | Fever;  Rash;  Liver function damage;  Mastoiditis; Interstitial lung disease (mild); | Mild loss  (20–40 dB) | Conjunctivitis;  Papillitis (mild) | Thickened meninges;  Sterile meningitis;  Developmental delay | Myalgia;  Arthralgia;  Arthritis (JADAS27: 8.3) |
| **P2**  **Post** | Pred+Cana | 16 M |  | 16 M | WBC (12.01)  CRP (22)  ESR (16)  Hb (115) | | Normal | Normal  (-10–20 Db) | Papillitis (very mild) | Thickened meninges(alleviated);  CSF test normal;  developmental catch-up; | (JADAS27: 0) |
| **P3**  **Before** | Pred+MTX+TCZ; Pred+ etanercept; Pred+Ada+  Sirolimus; | 21M |  | 21 M | WBC (29.81)  CRP (78)  ESR (38)  Hb (84) | | Fever;  Rash;  Splenomegaly | Normal  (-10–20 Db) | Papillitis | Headache;  Seizure;  Ventriculomegaly; Thickened meninges; Hydrocephalus;  Sterile meningitis;  Developmental delay; | Myalgia;  Arthralgia;  Arthritis (JADAS27: 13.8);  Metaphysis  Osteopathy;  Patella enlargement; |
| **P3**  **Post** | Pred+MTX+Cana | 20 M |  | 20 M | WBC (8.9)  CRP (＜10)  ESR (9)  Hb (118) | | Normal | Normal  (-10–20 Db) | Papillitis (very mild) | Ventriculomegaly; Thickened meninges alleviated;  Hydrocephalus alleviated; CSF test normal; | (JADAS27: 0);  Metaphysis  Osteopathy (milder) |
| **P4**  **Before** | Pred+MTX  NSAIDs | 3M |  | 3 M | WBC (27.08)  CRP (73.6)  ESR (55)  Hb (111) | | Fever;  Rash;  Splenomegaly;  Hepatomegaly;  Lymphadenovarix | Normal  (-10–20 Db) | Normal | Headache;  Ventriculomegaly; Thickened meninges; Hydrocephalus;  Sterile meningitis; Developmental delay; | Myalgia;  Arthralgia;  Arthritis (JADAS27: 12.5);  Patella enlargement |
| **P4**  **Post** | Pred+MTX+Cana | 11 M |  | 11M | WBC (9.28)  CRP (＜10)  ESR (12)  Hb (130) | | Normal | Normal  (-10–20 Db) | Normal | Ventriculomegaly; Thickened meninges alleviated;  Hydrocephalus alleviated; CSF test normal | (JADAS27: 0) |
| **P13 Before** | NSAIDs+ sirolimus+  IHN | 4 M |  | 4 M | WBC (37.5)  CRP (72)  ESR (61)  Hb (102) | | Fever;  Rash | Mild loss  (20–40 Db) | Normal | Normal | Normal |
| **P13**  **Post** | Cana | 15 M |  | 15 M | WBC (8.2)  CRP (＜10)  ESR (8)  Hb (122) | | Normal | Normal  (-10–20 Db) | Normal | Normal | Normal |
| **P14# before** | Pred+MTX+Ada | 14 M |  | 14 M | WBC (14.5)  CRP (66)  ESR (75)  Hb (98) | | Fever;  Rash;  Splenomegaly;  Hepatomegaly;  Lymphadenovarix;  Interstitial lung disease (mild); | Normal  (-10–20 dB) | Normal | Normal | Myalgia;  Arthralgia;  Arthritis (JADAS27: 25.5) |
| **P14#**  **Post** | Pred+MTX  +Cana | 10 M |  | 10 M | WBC (5.61)  CRP (＜10)  ESR (3)  Hb (127) | | erythema nodosum;  Interstitial lung disease (very mild) | Normal  (-10–20 dB) | Normal | Normal | JADAS27: 1 |
| **P15 Before** | Pred+TCZ; Pred+IFX;  Pred+Ada | 18 M |  | 18 M | WBC (8.9)  CRP (34)  ESR (39)  Hb (105) | | Fever;  Rash | Normal  (-10–20 dB) | Normal | Thickened meninges | Myalgia;  Arthralgia;  Arthritis (JADAS27: 6.9); |
| **P15**  **Post** | Cana | 8 M |  | 8 M | WBC (6.1)  CRP (＜10)  ESR (5)  Hb (125) | | Normal | Normal  (-10–20 dB) | Normal | Thickened meninges alleviated | JADAS27: 0 |
| **P16**  **Before** | Pred+  etanercept+  MTX; Pred+ MMF | 36M |  | 36 M | WBC (17.5)  CRP (92)  ESR (99)  Hb (95) | | Fever;  Rash;  Splenomegaly;  Hepatomegaly;  Lymphadenovarix; | Normal  (-10–20 dB) | Normal | Normal | Myalgia;  Arthralgia;  Arthritis (JADAS27: 17.9); |
| **P16**  **Post** | Pred+THD+MTX+Cana | 11 M |  | 11 M | WBC (6.4)  CRP (＜10)  ESR (6)  Hb (121) | | Normal | Normal  (-10–20 dB) | Normal | Normal | JADAS27: 2 |
| **P20**  **Before** | Ibuprofen | 9 M |  | 9 M | WBC (16.04)  CRP (77)  ESR (26)  Hb (100) | | Rash;  Fever | Normal  (-10–20 dB) | Normal | Normal | Myalgia;  Arthralgia;  Arthritis (JADAS27: 6.6) |
| **P20**  **Post** |  | 14 M |  | 14 M | WBC (7.9)  CRP (＜10)  ESR (2)  Hb (127) | | Normal | Normal  (-10–20 dB) | Normal | Normal | JADAS27: 0 |
| **P21**  **Before** | NSAIDS | 9 M |  | 9 M | WBC (20.1)  CRP (56)  ESR (46)  Hb (112) | | Rash | Normal  (-10–20 dB) | Normal | Normal | Myalgia;  Arthralgia;  Arthritis (JADAS27: 6.6) |
| **P21**  **Post** |  | 14 M |  | 14 M | WBC (7.21)  CRP (＜10)  ESR (5)  Hb (131) | | Normal | Normal  (-10–20 dB) | Normal | Normal | JADAS27: 0 |
| **P9 Post** | Pred+  NSAIDs+  THD+Cana | Lost |  | Lost | Lost | | Lost | Lost | Lost | Lost | Lost |

Before: before Canakinumb treatment; Post: post Canakinumb treatment. P2*: The inflammatory markers for P2 were decreased and were little above normal range; Afterwards, the intervals of Canakinumb administration were reduced to 4 weeks. P14#: P14 still had erythema nodosum before and post Canakinumb treatment.

M: month; Pred: prednisone; TCZ: tocilizumab; IFX: Infliximab; Ada: adalimumab; MMF: mycophenolate mofetil; Cana: Canakinumab; THD: thalidomide; MTX: methotrexate; NS: not sure; NSAIDs: nonsteroidal anti-inflammatory drugs; CNS: central nervous system; CSF: cerebrospinal fluid.

WBC () = WBC×10^9^/L. The units of CRP, ESR, and Hb are md/dL, mm/h, and g/L, respectively.

Table S6**.** Treatment and outcomes of patients not receiving Canakinumab

| Patients | Treatment strategy | Treatment course | Follow-up time | Outcome (last visit) |
| --- | --- | --- | --- | --- |
| P5 | Pred+THD+MTX+ Sirolimus | 22 M | 22 M | Fever daily; arthritis worse (JADAS27: 42); developmental delay; severe rash; high inflammatory markers; headache |
| P6 | Pred+MTX | 30 M | 30 M | Fever; arthritis worse (JADAS27: 23); developmental delay; severe rash; high inflammatory markers; headache |
| P7 | Pred+THD+MTX | Lost | Lost | Lost to follow-up |
| P8 | Pred+THD | Lost | Lost | Lost to follow-up |
| P10 | Pred+THD | Lost | Lost | Lost to follow-up |
| P11 | Pred+THD | Lost | Lost | Lost to follow-up |
| P12 | Pred+THD | Lost | Lost | Lost to follow-up |
| P17 | Pred+THD | 27 M | 27 M | No fever; normal inflammatory markers; occasional rash; sensorineural hearing loss alleviated |
| P18 | Pred+THD+MTX | 8 M | 8 M | Fluctuant inflammatory markers; severe rash; occasional fever; arthritis alleviated (JADAS27: 8.4) |
| P22 | No treatment | 21 M | 21 M | Rash; hearing loss |
| P23 | ibuprofen | 17 M | 17 M | No improvement |
| P24-26 | No treatment | 17 M | 17 M | No improvement |
| P27 | Tranilast+colchicine | 18 M | 18 M | Occasional fever; rash; fluctuant inflammatory markers |
| P28 | No treatment | 5 M | 5 M | No improvement |
| P29 | Pred+TCZ+MTX | 26 M | 26 M | Occasional rash; normal inflammatory markers; arthritis alleviated (JADAS27: 3.4) |
| P19 | Pred+ ibuprofen | 24 M | 24 M | Fever; high inflammatory markers |
| P30 | Tranilast | 22 M | 22 M | Severe rash; high inflammatory markers |

M: month; Pred: prednisone; TCZ: tocilizumab; IFX: Infliximab; Ada: adalimumab; MMF: mycophenolate mofetil; Cana: Canakinumab; THD: thalidomide; MTX: methotrexate.

Fig. S1 Pedigree of 25 families, 30 patients; 30 patients are from 25 families; F: family; P: patient;

Family 20 includes patient 20, 21 and 22; Family 21 includes patient 23, 24, 25 and 26;
